# A Practical Treatise on Diseases of the Skin

**Published:** 1881-03

**Authors:** 


### BIBLIOGRAPHICAL.

A Practical Treatise on Diseases of the Skin. By Louis A. Duhring, M.D., Professor of Diseases of the Skin in the Hospital of the University of Pennsylvania, etc. Second edition—revised and enlarged. J. B. Lippincott & Co., Philadelphia. 1881.

This is the latest edition of that admirable work of Professor Duhring on diseases of the skin, and requires no commendation from us. The merit of the author and the value of his works have long since been recognized and appreciated by the public. At best the study of the diseases of the skin is a troublesome thing, but the classification and arrangement of Prof. Duhring relieves the student of much difficulty in this respect. No better text-book on this subject can be found in the library of the specialist on diseases of the skin.

